# Variable Strength in Thoron Interference for a Diffusion-Type Radon Monitor Depending on Ventilation of the Outer Air

**DOI:** 10.3390/ijerph17030974

**Published:** 2020-02-04

**Authors:** Yasutaka Omori, Michikuni Shimo, Miroslaw Janik, Tetsuo Ishikawa, Hidenori Yonehara

**Affiliations:** 1 Department of Radiation Physics and Chemistry, Fukushima Medical University, 1 Hikarigaoka, Fukushima 960-1295, Japan; isikawat@fmu.ac.jp; 2 Center for Advanced Radiation Emergency Medicine, National Institutes for Quantum and Radiological Science and Technology, 4-9-1 Anagawa, Inage-ku, Chiba 263-8555, Japan; janik.miroslaw@qst.go.jp; 3 Fujita Health University, 1-98 Dengakugakubo, Kutsukake, Toyoake, Aichi 470-1192, Japan; shimomichikuni@sf.commufa.jp; 4 Nuclear Safety Research Association, 5-18-7 Shimbashi, Minato-ku, Tokyo 105-0004, Japan; yonehara@nsra.or.jp

**Keywords:** air exchange, diffusion, filter, infiltration, passive monitor, radon, thoron interference, ventilation

## Abstract

Thoron interference in radon measurements using passive diffusion radon detectors/monitors is a crucial problem when it comes to assessing the internal exposure to radon precisely. The present study reported, as one of the potential factors, the effects of air flow conditions on changes in thoron interference. Rates of thoron infiltration (as thoron interference) into the diffusion chamber of the monitor were evaluated. The temporal variation was obtained based on measurements of the underfloor space of a Japanese wooden dwelling using a diffusion-type radon monitor, a reference radon monitor which was not affected by thoron interference, and a thoron monitor. The thoron infiltration rate for the diffusion-type monitor varied from 0% to 20%. In particular, it appeared to increase when ventilation of the underfloor space air was forced. The variable thoron infiltration rate, with respect to ventilation strength, implied that not only a diffusive process, but also an advective process, played a major role in air exchange between the diffusion chamber of the monitor and the outer air. When an exposure room is characterized by the frequent variation in air ventilation, a variable thoron response is considered to occur in radon–thoron discriminative detectors, in which only diffusive entry is employed as a mechanism for the discrimination of radon and thoron.

## 1. Introduction

The inhalation of radon (^222^Rn, Rn; half-life: 3.8 days) is one of the most important pathways causing prolonged exposure from natural radiation sources [1]. Radon detectors/monitors with diffusion chambers, such as those with solid-state nuclear track detectors and a pulse-ionization chamber, have been used to measure radon concentration and assess the dose of radon. However, in some cases, the measured values should be analyzed carefully because of their overestimation, caused by the naturally coexisting radon isotope thoron (^220^Rn, Tn), which has a half-life of 55.6 s (i.e., thoron interference). Tokonami et al. [2] reported on the thoron interference in radon measurements in thoron-prone areas of China, which led them to reevaluate the internal dose of radon, and which later led Doi et al. [3] and Akiba et al. [4] to reconsider possible risks due to radon exposure. To avoid thoron interference, a number of radon-thoron discriminative detectors have been developed (e.g., [5,6,7,8]) and used in recent national and regional surveys (e.g., [9,10,11,12,13,14,15]).

Thoron interference has been reported in previous studies (e.g., [16,17,18,19,20,21]) for integration type radon detectors and continuous measurement type radon monitors. In these detectors/monitors, natural air exchange (i.e., diffusion) is a mechanism which works by introducing air into the diffusion chamber. Tokonami et al. [16] examined the former type of detectors, which were composed of radon detectors and radon-thoron discriminative detectors. Their experiment revealed that rates of thoron infiltration into the diffusion chamber of the detectors varied from 0% to 100%. That is, radon concentration was overestimated by a factor of up to two when radon and thoron concentrations were the same as each other. Ishikawa [17] carried out a different experiment, in which he investigated a pulse-ionization chamber monitor, the AlphaGUARD PQ2000Pro (Genitron Instruments, GmbH (Frankfurt, Germany)—now Bertin Technologies (Montigny-le-Bretonneux, France)); this monitor is widely used in environmental studies (see, for example, [22,23,24]). In Ishikawa’s study [17], the chamber monitor was exposed to radon-bearing and thoron-bearing air in a calibration chamber constructed at the Environmental Measurements Laboratory in New York, and a thoron infiltration rate of about 10% was seen. A similar result was obtained by Kochowska et al. [18] and Sumesh et al. [19]. Based on all of these studies, when such detectors/monitors without the function of discrimination between radon and thoron are used, overestimation of radon concentration should be presumed in surveys conducted in, for instance, thoron-prone areas.

Experiments on thoron interference in radon measurements have been carried out under controlled environments, such as in calibration chambers. However, in real (natural) environments, rates of thoron infiltration into the diffusion chamber of detectors and monitors may vary with time and the environmental conditions surrounding them (e.g., air temperature, humidity, and circulation in a room). This is connected to the properties of filters and sponges covering the inlet of detectors/monitors to prevent ambient aerosols infiltrating. A possible increase in the thoron infiltration rate with an increase in air temperature is inferred from the finding that the permeability of radon through a membrane filter increased with membrane temperature (e.g., [25]). Sorimachi et al. [26] reported a decreasing thoron infiltration rate with an increasing relative humidity due to the adsorption of air water into the filter/sponge. The present study reported that the thoron interference in the radon measurements varied depending on the ventilation of the air surrounding the diffusion-type radon monitor.

## 2. Materials and Methods

The present study examined thoron interference in radon concentrations indicated by a diffusion-type radon monitor. Natural air exchange (i.e., diffusion) is a mechanism which works by introducing air through a filter into the diffusion chamber of the monitor. Under exposure to the mixed air of radon and thoron, radon and thoron concentrations inside and outside of the diffusion chamber of the monitor satisfy the following derivative equations with respect to time *t*

(1) $$\frac{dC_{\mathrm{Rn},\;\mathrm{in}}}{dt}=-\lambda_{\mathrm{Rn}}C_{\mathrm{Rn},\;\mathrm{in}}+\gamma \left(C_{\mathrm{Rn},\;\mathrm{out}}-C_{\mathrm{Rn},\mathrm{in}}\right),$$

(2) $$\frac{dC_{\mathrm{Tn},\;\mathrm{in}}}{dt}=-\lambda_{\mathrm{Tn}}C_{\mathrm{Tn},\;\mathrm{in}}+\gamma \left(C_{\mathrm{Tn},\;\mathrm{out}}-C_{\mathrm{Tn},\mathrm{in}}\right),$$

where *C*_Rn, in_, *C*_Rn, out_, *C*_Tn, in_, and *C*_Tn, out_ are radon and thoron concentrations inside and outside of the diffusion chamber of the monitor, respectively, *λ*_Rn_ and *λ*_Tn_ are the decay constants of radon and thoron, respectively, and *γ* is air exchange rate [19,27]. As seen in Equations (1) and (2), thoron interference is connected to the rate of air exchange through a filter.

The rate of thoron infiltration into the diffusion chamber of the radon monitor as thoron interference in radon measurement was examined in comparison to radon concentrations (*C*_Rn-T_) possibly affected by thoron interference, reference radon concentrations (*C*_Rn-R_) not affected by thoron interference, and thoron concentrations (*C*_Tn, out_). Similar to the studies conducted by Ishikawa [17] and Sumesh et al. [19], differences in *C*_Rn-T_ and *C*_Rn-R_ are regarded as values enhanced by thoron infiltration into the diffusion chamber. Hence, thoron infiltration rate (*R*) can be formulated as

(3) $$R\;\left(\%\right)=\frac{C_{\mathrm{Rn}-T}\;-\;C_{\mathrm{Rn}-R}}{C_{\mathrm{Tn},\;\mathrm{out}}}\times 100.$$

The radon monitors used in the present study were AlphaGUARD PQ2000Pro pulse-ionization chambers, which are the same as those used in the aforementioned studies [17,19]. The AlphaGUARD monitors can run in diffusion or flow mode. The AlphaGUARD monitor in the diffusion mode was used as a diffusion-type radon monitor for investigating thoron interference—that is, the radon monitor in the diffusion mode measured *C*_Rn-T_. The sampling interval was 1 h. In contrast, the other AlphaGUARD monitor was set to flow mode to run as a reference radon monitor with radon–thoron discrimination (e.g., [28]). The air was pumped with a flow rate of 1.0 × 10^−4^ m^3^ min^−1^ into the radon monitor through a polyolefin tube (Tygon LMT-55 SCFJ00033, Saint-Gobain K.K., Tokyo, Japan), and passed through a glass fiber filter (Whatman GF/F, 47 mm in diameter, GE Healthcare Japan Corporation, Tokyo, Japan). The length of the tube was adjusted so that the travel time of the sampled air was 10 min. This is equivalent to about 10 half-lives of thoron, so that thoron interference in the detection unit would be negligible and the radon monitor in flow mode would be able to measure *C*_Rn-R_. The radon concentration was measured every 10 min and was converted into a one hour averaged value. These AlphaGUARD radon monitors were calibrated internally using a radon calibration chamber established in the National Institute of Radiological Sciences (NIRS; now a part of the National Institutes for Quantum and Radiological Science and Technology, Japan). The properties of the radon monitors are summarized in Table 1.

Thoron concentration (*C*_Tn, out_) was measured by the RTM2200 (SARAD GmbH, Dresden, Germany). This thoron monitor used alpha spectrometry with a semiconductor detector (Table 1), which had been calibrated against a reference instrument in the German Federal Office for Radiation Protection Bundesamt für Strahlenschutz accredited by the Physikalisch Technische Bundesanstalt. The filtered air was pumped into the detection unit with a flow rate of 3.0 × 10^−4^ m^3^ min^−1^ and thoron concentration was measured every hour.

Simultaneous measurement using these three types of radon and thoron monitors was carried out in a Japanese dwelling located in Gifu Prefecture. The selected dwelling was a two-story wooden structure with an underfloor space, a characteristic which is common to many residences in Japan. The underfloor space was approximately 70 cm in height and above the ground surface (there was no building material covering the surface), and the air in the space was ventilated naturally from openings or by a forced fan exhaust system. The radon and thoron monitors were installed in the underfloor space. These monitors were located 22 cm above the ground surface and 200 cm away from the fans’ covering openings. The altitude was almost the same between these monitors and the fan. When the forced-fan exhaust system was run, the wind speed increased from around 0.03 to 0.12 m s^−1^ in front of the fan, which was measured by a handheld directional anemometer (Model 6531, KANOMAX Japan Incorporated, Osaka, Japan).

## 3. Results and Discussion

**Figure 1:**
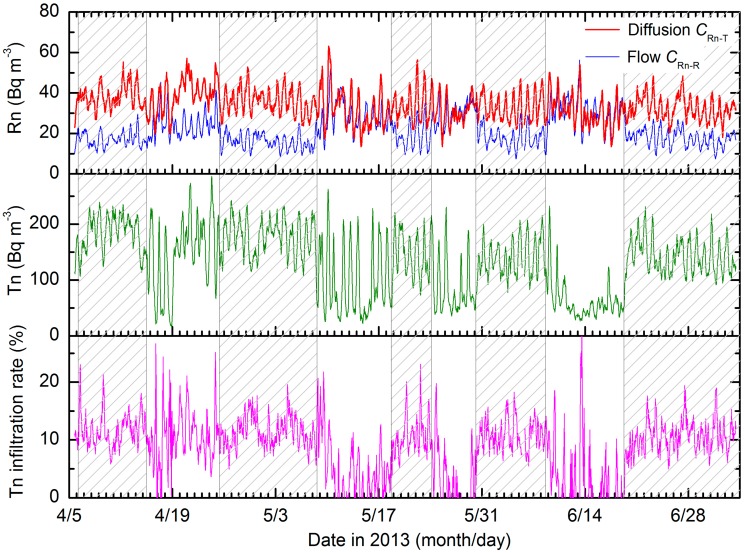
The temporal variations in radon concentrations in diffusion and flow modes and thoron concentration, together with the thoron infiltration rate calculated from Equation (3) in the text. During the periods indicated by shaded areas, a forced fan exhaust system was run to ventilate the underfloor space air. Thoron infiltration rates which had negative values were excluded from the figure.

Concentrations of radon (*C*_Rn__-T_ and *C*_Rn-R_) and thoron (*C*_Tn, out_) in the underfloor space were analyzed from 5 April to 4 July, 2013. Figure 1 presents temporal variations in radon concentrations measured with the radon monitors in the diffusion and flow modes together with thoron concentration. A five hour moving average was applied to smooth their temporal variations. It is noted again that radon concentrations measured in the diffusion mode were the ones (*C*_Rn-T_) possibly affected by thoron interference, whereas radon concentrations measured in the flow mode were reference radon concentrations (*C*_Rn-R_) not affected by thoron interference. As seen in Figure 1, the forced-fan exhaust system was run to ventilate the underfloor space air repeatedly for around 10 days, and totally during about 60% of the measurement period. The *C*_Rn-T_ values varied in the range of 20–50 Bq m^−3^, and they were nearly constant throughout the observation period. In contrast, the *C*_Rn-R_ values varied in the range of 10–40 Bq m^−3^, and they were lower when the forced-fan exhaust system was run, because the underfloor space air was replaced with fresh outdoor air with a lower radon concentration. Specifically, the differences in these two quantities depended on the ventilation strength of the underfloor space air. Significant differences were found during the forced ventilation periods, whereas only small differences were found during the natural ventilation periods.

Figure 1 also shows the dependence of thoron concentration on the ventilation strength in the underfloor space air. Thoron concentrations tended to be higher and have weaker diurnal variations during the forced ventilation periods compared to those obtained during the natural ventilation periods. The exact reason why thoron concentrations were higher during the forced ventilation periods is unclear, but thoron exhalation from the ground surface may increase due to induced pressure gradients which can draw soil gases (i.e., the pumping effect), and some thoron atoms exhaled may reach the thoron monitor without significant radioactive decay.

**Figure 2:**
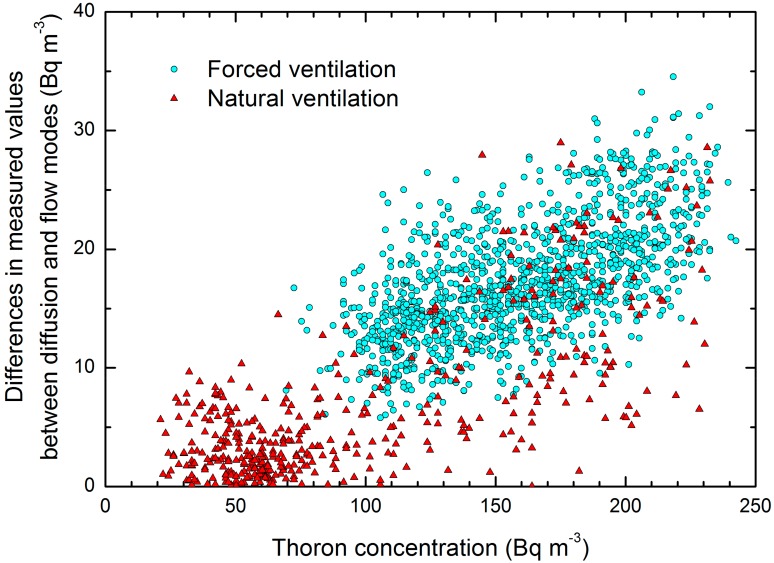
A scatter plot of differences in measured values between the diffusion and flow modes against thoron concentrations during the natural ventilation (red triangles) and forced ventilation (light blue circles) periods. The differences with negative values and those from between 19 and 25 April, when strong ventilation seems to have occurred as inferred from the measured thoron concentrations, were excluded from the figure.

The changes in the difference in the values between the two radon monitors were partially attributed to thoron interference, due to the changes in thoron concentration in the underfloor space air. Figure 2 presents a scatter plot of differences in the radon concentrations (i.e., *C*_Rn-T_ minus *C*_Rn-R_) in the diffusion and flow modes, against thoron concentrations during the natural ventilation and forced ventilation periods. The differences with negative values, and those from between 19 and 25 April, when strong ventilation seems to have occurred, as inferred from the measured thoron concentrations, were excluded for the analysis. There was a positive correlation between them during the forced ventilation periods; thoron infiltrated into the diffusion chamber and disturbed radon measurement in the diffusion mode. However, there did not appear to be a clear correlation during the natural ventilation periods. The lack of correlation can probably be attributed to the narrower range of thoron concentrations; thoron concentration was mostly distributed between 20 and 100 Bq m^-3^ during the natural ventilation periods. The lack of correlation may also be attributed to natural gas circulation in the underfloor space; this can be influenced by outdoor wind field (intensity and direction) causing pressurization and depressurization in indoor and underfloor spaces.

**Figure 3:**
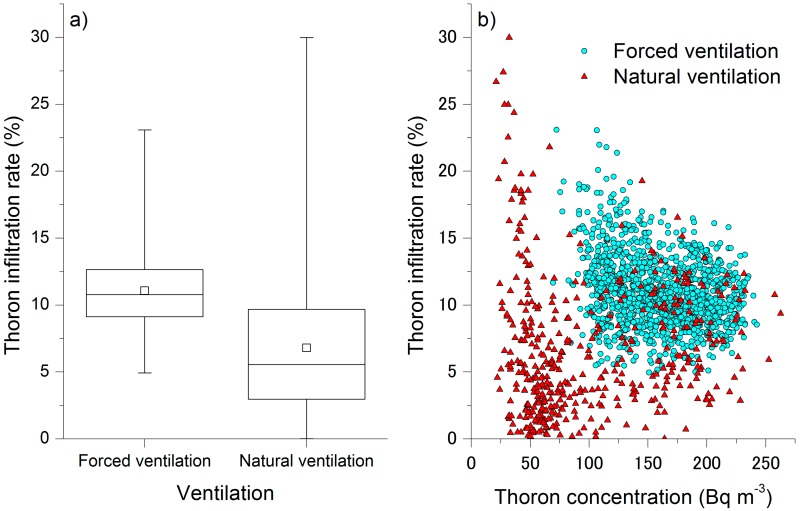
Box and whisker plots of the thoron infiltration rate with respect to ventilation strength (**a**), and a scatter plot of thoron infiltration rates against thoron concentrations during the natural ventilation (red triangles) and forced ventilation (light blue circles) periods (**b**). The squares in the boxes represent the arithmetic mean, and the whiskers represent the range from minimum to maximum rates. Thoron infiltration rates between 19 and 25 April were excluded for the analysis.

The difference could not be caused only by the change in thoron concentration. Figure 1 presents a temporal variation in thoron infiltration rate, calculated from the *C*_Rn-T_, *C*_Rn-R_, and C_Tn, out_ values based on Equation (3). Thoron infiltration rate fluctuated around 10% and its variation was less during the forced ventilation periods. In contrast, although the rate was 10% or above some of the time, the rate of thoron infiltration into the diffusion chamber of the monitor was only a few percent or zero during the natural ventilation periods. The box and whisker plots drawn in Figure 3a support the finding that there was a clear difference with respect to ventilation strength. The median values of the thoron infiltration rate were 5.5% and 11% in natural and forced ventilation, respectively. Figure 3b does not confirm that the change in the thoron infiltration rate was caused by thoron concentration. These results indicate that the thoron infiltration rate was partially constrained by the ventilation strength of the air surrounding the radon monitor, and that the change of the rate affected the radon measurements. In the present study, thoron infiltration rates obtained during natural ventilation and forced ventilation periods were comparable to other reported experimental results (5–10%) in radon and thoron exposure [17,18,19].

Furthermore, the findings of the present study point out the process of air exchange in the diffusion-type monitor. Previous studies [6,29,30] expressed the air exchange rate as follows

(4) $$\gamma =\frac{D_{p}A}{dV},$$

where *D*_p_ is the diffusion coefficient in a porous medium such as a filter paper, *d* is the thickness of the medium, *A* is the opening area and *V* is the volume of the monitor. The diffusion coefficient depends on air temperature and the porosity and tortuosity of the medium. In the present study, thoron infiltration rate, which is linked to air exchange rate in the radon monitor, was affected by the ventilation strength of the underfloor space air. This implies that the migration in the porous media is constrained not only by a diffusive process but also by an advective one, so that Equation (4) may be rewritten as follows

(5) $$\gamma =\frac{A}{V}\left(\frac{D_{p}}{d}+u\right),$$

where *u* is air velocity induced by pressure difference in the medium. This relation can also be assumed in integration-type radon–thoron discriminative detectors, in which only diffusive entry is employed as a mechanism for the discrimination of radon and thoron (e.g., [8,26,30]). Thus, the results discussed above suggest that, when an exposure room is characterized by a frequent variation in air ventilation, a variable thoron response is considered to occur in those detectors.

Unlike the previous studies (e.g., [17,18,19,20]) conducted using calibration chambers, the present study was carried out in the natural environment of the underfloor space of a Japanese dwelling. Due to this, thoron concentration was relatively low, 10–100 Bq m^-3^, and fluctuated as diurnal variations in the present study. These factors may have influenced part of the present results, for instance, the wide distribution of thoron infiltration rate. In a future study, a change in thoron infiltration rate with respect to ventilation strength would be examined based on measurements using a thoron calibration chamber, in which thoron concentration can be controlled to be constant at a higher level, around 1000–10,000 Bq m^−3^ (e.g., [31,32]).

## 4. Conclusions

In the present paper, a diffusion-type radon monitor was used to examine thoron interference in radon measurements in the underfloor space of a Japanese wooden dwelling. The result showed that the thoron infiltration rate as thoron interference varied and was about 5.5% and 11% in natural ventilation and forced ventilation of the underfloor air, respectively. This difference might have been caused by the change in advective process during infiltration in air exchange between the diffusion chamber of the radon monitor and the outer air. The results suggest that, when an exposure room is characterized by frequent variations in air ventilation, variable thoron response is considered to occur in radon–thoron discriminative detectors, in which only diffusive entry is employed as a mechanism for the discrimination of radon and thoron.

## Figures and Tables

**Table 1:** Properties of the radon and thoron monitors used in the present study.

Instrument	Detection Principle	Measurement Mode	Measurement Cycle	Measurand (Parameter in Equation (3))
AlphaGUARD-1	Pulse-ionization chamber	Diffusion	1 h	Radon (*C*_Rn-T_) ^1^
AlphaGUARD-2	Pulse-ionization chamber	Flow	10 min	Radon (*C*_Rn-R_) ^2^
RTM2200	Alpha spectrometry with a semiconductor detector	Flow	1 h	Thoron (C_Tn, out_)

^1^ The radon concentration possibly affected by thoron interference. ^2^ The reference radon concentration not affected by thoron interference.
